# A UCP1-IRES-Cre Knock-In Mouse Enables Specific Brown Adipocyte Targeting Without CNS Off-Target Expression

**DOI:** 10.21203/rs.3.rs-9752374/v1

**Published:** 2026-06-17

**Authors:** Joshua Meyer, Jaclyn Williams, Jennifer Bailey, Ji Suk Chang, Carrie M. Elks, Heike Münzberg

**Affiliations:** Pennington Biomedical Research Center; Pennington Biomedical Research Center; Pennington Biomedical Research Center; Pennington Biomedical Research Center; Pennington Biomedical Research Center; Pennington Biomedical Research Center

**Keywords:** UCP1, adipose tissue, IRES-Cre, BAC-transgenics, hypothalamus

## Abstract

Uncoupling protein 1 (UCP1) is predominantly expressed in brown adipocytes, where it plays a central role in thermogenesis and energy metabolism. BAC-transgenic UCP1-Cre mouse lines have been widely used to target thermogenic adipocytes, but ectopic Cre activity in the hypothalamus and peripheral organs has raised concerns regarding specificity. Here, we characterize a novel knock-in UCP1-IRES-Cre mouse model that drives Cre expression from the endogenous UCP1 promoter. UCP1-IRES-Cre mice were crossed with Ai14 reporter mice to visualize Cre-dependent tdTomato expression across adipose depots, brain regions, and peripheral tissues and compared with UCP1 protein localization. Reporter expression in male and female UCP1-IRES-Cre mice closely mirrored endogenous UCP1 expression in brown and white adipose tissue depots, with substantially reduced ectopic activity compared with previously reported BAC-transgenic models. Minimal reporter expression was observed in the hypothalamus and peripheral organs, although robust reporter expression was detected in the choroid plexus despite absent UCP1 protein staining. White adipose depots exhibited broader reporter labeling than UCP1 immunoreactivity, consistent with transient or prior UCP1 promoter activation during development or adult life. Together, these findings demonstrate that the UCP1-IRES-Cre model faithfully recapitulates endogenous UCP1 expression while minimizing off-target Cre activity, providing a knock-in alternative for genetic targeting of thermogenic adipocytes.

## 1. Introduction

Brown adipose tissue (BAT) is an important thermogenic and energy-expending tissue that is present in all mammals, including humans. Brown adipocytes are distinguished from energy-storing white adipocytes by their expression of uncoupling protein-1 (UCP1), a mitochondrial protein that bypasses ATP synthesis and instead releases energy as heat. UCP1 serves as a defining molecular marker for brown and beige adipocytes and their role in thermogenesis [1–3].

The bacterial artificial chromosome (BAC) Tg(UCP1-Cre)Evdr/J mouse line was generated to enable selective genetic manipulation of thermogenic brown adipocytes [4–7]. The Tg(UCP1-Cre)Evdr/J mouse strain has been widely adopted and accounts for approximately 79% of the Ucp1-driven entries in the Mouse Genome Informatics (MGI) database in 2025 [8]. This model was generated using a BAC containing the full Ucp1 genomic locus, with the Cre recombinase cassette inserted into the translational start site of Ucp1 [4].

Recently, validation studies of the BAC Tg(UCP1-Cre)Evdr/J mouse line have shown robust ectopic Cre activity in the central nervous system, including the hypothalamus, despite endogenous Ucp1 being at or near the limit of detection in the adult brain [8–11]. Cre activity has also been reported in peripheral tissues, including kidneys and adrenal gland, indicating that recombination driven by the transgene is not restricted to thermogenic adipose tissue [8, 9]. Recent genetic studies further suggested that the Tg(UCP1-Cre)Evdr/J line may harbor transgene-associated chromosomal rearrangements that could contribute to off-target recombination [8]. Together, these findings highlight a potential limitation of transgenic mouse models, particularly BAC-based constructs, which may be more susceptible to off-target recombination than knock-in strategies [12].

To address these limitations, the field has shifted toward knock-in strategies where Cre recombinase is driven by the endogenous promoter via an internal ribosome entry site (IRES). This approach is widely used for generating cell-specific Cre-driver lines, as it preserves endogenous promoter regulation. Here we report the generation and validation of a knock-in UCP1-IRES-Cre mouse model. To validate Cre activity, we generated UCP1-reporter mice and confirmed robust recombination in brown adipocytes, while verifying the lack of reporter activity in the brain and peripheral tissues previously reported to exhibit Cre activity in Tg(UCP1-Cre)Evdr/J mice [9–11].

## 2. Results

### 2.1 IRES-Cre-UCP1-reporter mice show similar distribution of UCP1 protein and reporter expression in iBAT and iWAT.

**Figure 1:**
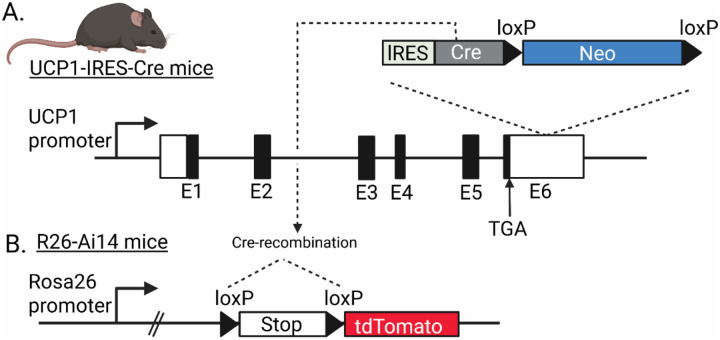
Generation of UCP1-IRES-Cre mice and experimental reporter mice. **A.** Schematic of the genetic modification used to generate UCP1-IRES-Cre mice. **B.** UCP1-IRES-Cre mice were crossed with R26-Ai14 reporter mice to visualize cells with active UCP1-IRES-Cre-mediated recombination. *IRES, inter-ribosomal-entry-site; UCP1, uncoupling protein 1*

**Figure 2:**
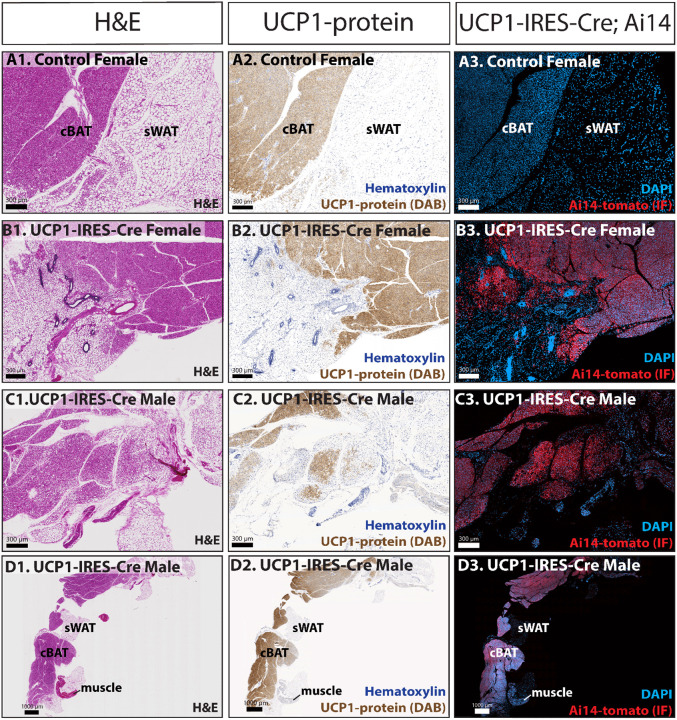
UCP1-reporter expression closely reflects UCP1-protein expression in iBAT. Paraffin-embedded 5 μm sections spanning the interscapular brown adipose tissue (iBAT) depot were stained with hematoxylin/eosin (H&E) (**A1-D1**), UCP1 peptide (DAB brown) with hematoxylin counterstain (blue) (**A2-D2**), or anti-RFP detection of Ai14-tdTomator reporter (red) with DAPI (blue) (**A3-D3**). **A1-C1:** Higher-magnification images from core BAT (cBAT) and surrounding WAT (sWAT) from a control female, a UCP1-IRES-cre female (B1-B3) and a UCP1-IRES-cre male (C1-C3). **D1-D3:** Low-magnification overview of the iBAT depot from a male UCP1-IRES-cre mouse. **A1-D1:** H&E staining distinguishes dense, multilocular brown adipocytes in cBAT from unilocular white adipocytes in sWAT. **A2-D2:** UCP1 protein labeling is prominent in cBAT, but absent in sWAT. **A3-D3:** Ai14-tdTomato is absent in control mice (A3), but robustly labels cBAT in UCP1-IRES-Cre mice in females (B3) and males (C3, D3). Note the higher DAPI signal density in cBAT compared to sWAT, reflecting differences in adipocyte size and density. *Scale bars are 300um (A-C) or 1000um (D1-D3)*.

We assessed the accurate expression of Cre activity from UCP1-IRES-Cre mice by crossing them with Ai14-tdTomato reporter mice (Fig. 1). Both males and females were analyzed for UCP1 protein and Cre-dependent tdTomato reporter expression. In core iBAT, UCP1-expressing brown adipocytes are easily distinguishable from the surrounding white adipocytes due to their increased cytoplasmic content in H&E images (Fig. 2, A1- D1) and UCP1 protein signal (Fig. 2, A2-D2). Cre signal overlapped in both male and female mice (Fig. 2, A3-D3) but was absent in controls. There was no difference in iBAT expression data between males and females. Thus, overall, this data demonstrates that Cre-induced tdTomato reporter expression corresponds with UCP1 expression.

Upon closer inspection of the transition zone from dense core iBAT to surrounding white adipocytes, often referred to as beige adipocytes, we found that many adipocytes lacking a UCP1 protein signal were positive for the tdTomato reporter signal. This discrepancy is consistent with prior UCP1 expression, indicating the presence of extensive “pre-beiged” adipocytes, e.g., during development or early adult life, which would induce irreversible tdTomato expression.

### 2.2. Cre expression is more widespread than adult UCP1 expression in iWAT.

**Figure 3:**
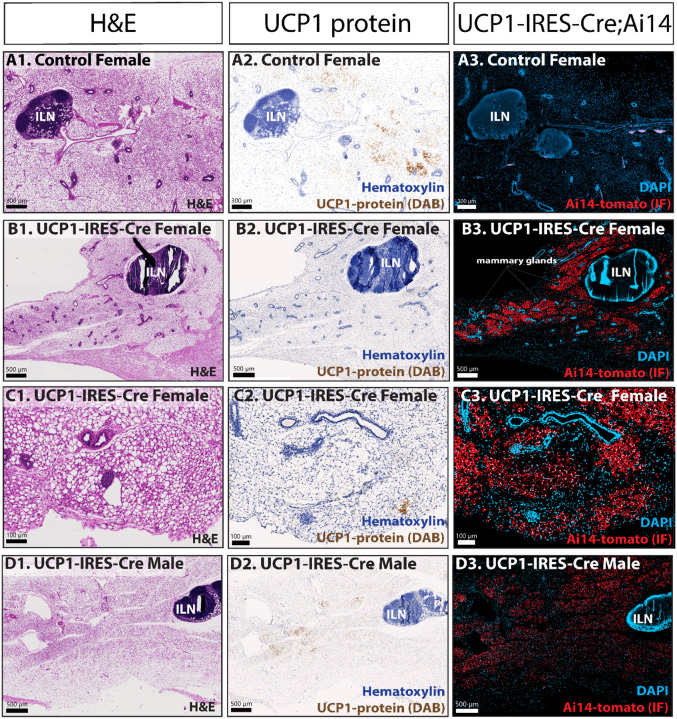
UCP1-reporter expression is more expansive than UCP1-protein expression in iWAT. Paraffin-embedded 5 μm sections of the interscapular white adipose tissue (iWAT) from female (**A,B,C**) and male (**D**) mice was processed for H&E staining (**A1-D1**), UCP1 peptide (DAB brown) with hematoxylin (blue)(**A2-D2**) or anti-RFP detection of Ai14-tdTomato (red) with DAPI (blue)(A3-D3). **A-D:** Images sampled near the inguinal lymph node (ILN) as an anatomical landmark (**A, B, D**). Panel C shows an example from the more distal inguinal region of iWAT. Note, the denser eosinophilic regions visible in H&E morphology are consistent with putative beige adipocyte clusters (“beige islands”). **A2-D2:**UCP1 protein expression was low or undetectable in iWAT across mice, including regions with increased cellular density and suggestive of beige islands. **A3-D3**: Ai14-tdTomato expression was absent in control mice but robustly detected in all UCP1-IRES-cre mice. Reporter-positive labeling in some cases overlapped with denser eosin-stained regions observed by H&E, despite minimal UCP1 peptide detection at the time of analysis, consistent with prior or transient UCP1 expression in these adipocytes. *Scale bars are 500um (Panel A, B, and D)) or 100um (C1-C3)*.

In the iWAT (Fig. 3), beige islands are known to exist, where clusters of adipocytes express UCP1 [13]. Interestingly, we found only few sites with UCP1 protein expression in this mouse line (Fig. 3, A2-D2), which was in striking contrast to the widespread tdTomato reporter signal (Fig. 3, A3-D3) in a pattern that mirrored the expected location of beige islands proximal to the iWAT lymph node and further down the inguinal extension of iWAT [13]. We observed more tdTomato-positive adipocytes than UCP1 immunopositive cells (Fig. 3, B2-C2). The tdTomato signal in adipocytes extended well beyond the region with detectable UCP1 protein, similar to what we observed in the iBAT transition zone. As expected, no tdTomato signal was detected in control iWAT (Fig. 3, A3), supporting the interpretation that tdTomato-positive adipocytes represent adipocytes with previous UCP! expression.

### 2.3 Lack of evidence for ectopic expression in the brain.

**Figure 4:**
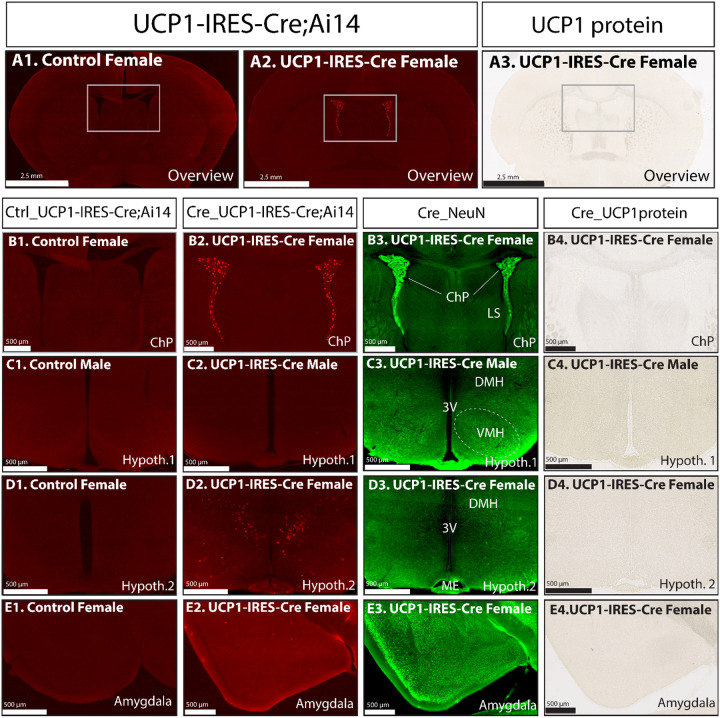
Minimal ectopic reporter expression in the brain of UCP1-IRES-Cre mice. Coronal 30 μm brain sections show endogenous reporter expression (Ai14-tdTomato, red) in control mice (**A1-E1**) or UCP1-IRES-cre mice (**A2-E2**). Adjacent sections were stained for the neuronal marker NeuN (green, **B3-E3**) or UCP1-peptide (**A3, B4-E4**). **A1-A2.** Low-magnification overview depicting the choroid plexus (ChP) shows no reporter signal in control mice (A1), while UCP1-ires-cre mice (**A2**) exhibit prominent tdTomato labeling in the ChP despite the absence of detectable UCP1 protein. **B1-B2** Higher-magnification images of the ChP confirming tdTomato labeling. **C1-D1 :** The hypothalamus showed no detectable reporter or UCP1 protein labeling in controls or UCP1-IRES-cre mice (**C2**). Rare tdTomato positive neurons were observed bilaterally in the caudal dorsomedial hypothalamus of UCP1-IRES-cre mice (**D2**). **E1-E2:** The amygdala showed no reporter expression in controls or UCP1-IRES-cre mice. *Scale bars are 2.5 mm (A1-A3) or 500 um (B1-E1)*. 3^rd^ ventricle (3V), median eminence (ME), choroid plexus (ChP), ventromedial hypothalamus (VMH), dorsomedial hypothalamus (DMH)

Because the Tg(UCP1-Cre)Evdr/J mouse model showed strong ectopic reporter expression in the hypothalamus and choroid plexus, we investigated the expression levels in control and UCP1-IRES-Cre mice (Fig. 4). Overall, there was minimal tdTomato expression in the brains of control and UCP1-IRES-Cre mice except for a prominent signal in the choroid plexus that was absent in control mice (Fig. 4, A1, B1), but strong in UCP1-IRES-Cre mice (A2, B2). We further verified that UCP1 protein is not expressed in the adult choroid plexus (Fig. 4, A3, B4), so this Cre-specific signal may be due to UCP1 expression during development or prior to tissue harvest, which would maintain reporter expression once Cre recombination occurs. There was no reporter signal in the ventromedial hypothalamus,(VMH) (Fig. 3, C1,C2), although in two female mice, we found a cluster of 20–30 reporter-positive neurons in the dorsomedial hypothalamus (DMH) (Fig. 4, D1, D2), while two other UCP1-IRES-Cre brains (one male and one female) exhibited 5–10 reporter-positive neurons. Finally, neither the control nor the UCP1-IRES-Cre mice exhibited tdTomato reporter signals or UCP1 protein expression in the amygdala (Fig. 4,E1-E2, E4). However, the few sites with reporter signals were observed in only UCP1-IRES-Cre mice and indicated some expression in select choroid plexus cells at some point during the life of the mice. We additionally evaluated tdTomato reporter expression in the parafascicular thalamic nucleus (PaF) as a direct comparison with previous reports in Tg(UCP1-Cre)Evdr/J mice (Supplemental Fig. 1) [9]. We observed tdTomato expression in half (n = 2) of the UCP1-IRES-Cre brains (n = 4) (Supplemental Fig. 1). Interestingly, the same two animals with more localized tdTomato expression in the DMH (Fig. 4) also showed expression in the PaF.

### 2.4 Minimal or no ectopic expression in heart, liver, pancreas, kidney/adrenal tissue

**Figure 5:**
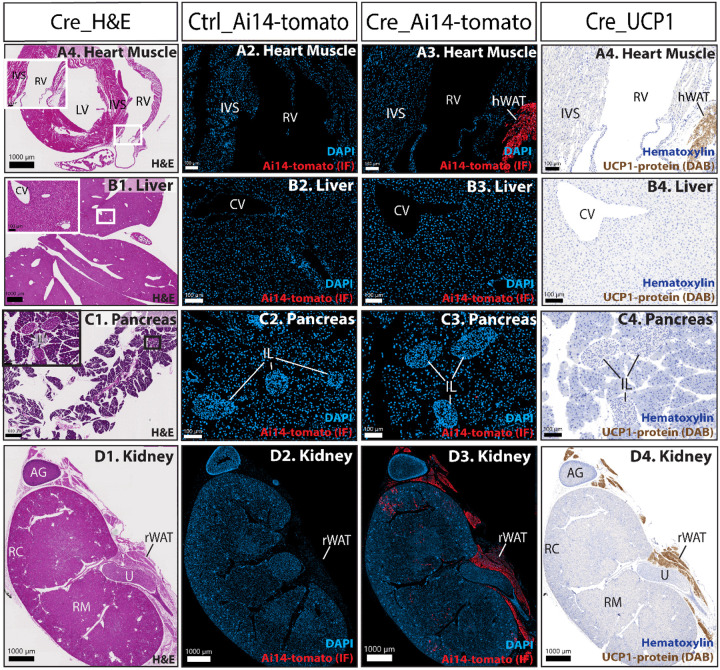
Lack of UCP1-reporter expression in muscle, liver, kidney, pancreas. Paraffin-embedded 5 μm sections of heart (**A1-A4**), liver (**B1-B4**), pancreas (**C1-C4**), and kidney (**D1-D4**) were processed for H&E stain (**A1-D1**), anti-RFP detection of Ai-tdTomato (red) with DAPI (**A2-D2**) or UCP1-DAB protein stain with hematoxylin (**A3-D3**). Low magnification overviews show characteristic anatomical landmarks for each tissue (heart: IVS, LV, RV; liver: CVs; pancreas: ILs; kidney RC, RM, U, AG) with boxed regions shown at higher magnification insets. Ai14-tdTomato reporter expression was absent in cardiac muscle, liver, pancreas and kidney parenchyma of both controls and UCP1-IRES-cre mice, consistent with the lack of detectable UCP1 protein in these tissues. In contrast, adjacent adipose tissue depots exhibited strong reporter expression such as heart-associated WAT (hWAT, **A2**) and renal WAT (rWAT, **D3**) showed prominent Ai-tdTomato labeling together with detectable UCP1 protein (**D4**), consistent with their classification as beige fat depots. Rare scattered tdTomato-positive cells were consistently observed in the kidney of all UCP1-IRES-cre mi ce (**D3**), whereas no signals were observed in the adrenal gland or urether. *interventricular septum (IVS), left and right ventricles (LV, RV), central veins (CV), islets of Langerhans (IL), renal medulla (RM) and renal cortex (RC), ureter (U), adrenal gland (AG)*.

We analyzed heart, liver, pancreas, and kidney tissue for UCP1 and reporter expression, UCP1 was found in adipocytes located around those organs, specifically heart, kidney and adrenal glands (Fig. 5). In the myocardium, we did not observe UCP1 reporter expression; however, in the surrounding heart-associated white adipose tissue (hWAT) tdTomato reporter expression (Fig. 5, A3) corresponds with UCP1 protein expression (Fig. 5, A4). Across all liver (Fig. 5, B1-B4), pancreas (Fig. 5, C1-C4) and adrenal tissues (Fig. 5, D1-D4), neither tdTomato nor UCP1 protein expression was observed in Cre-positive or control mice, supporting minimal off target reporter activity. In the kidney (Fig. 5, D1-D4), a noticeable but scattered tdTomato signal was observed within the renal medulla and cortex. The surrounding renal adipose tissue showed tdTomato signals (Fig. 5, D3) as a truthful representation of UCP1 protein-positive adipocytes (Fig. 5, D4), while some “pre-beiged” adipocytes with tdTomato signal but lacking UCP1 protein expression were also identified. Again, we found large portions of pre-beiged tdTomato-positive adipocytes that lacked a UCP1-protein expression, like the pattern found in iBAT and iWAT. Importantly, all tissues analyzed in this group (experimental and control) showed minimal leakiness of IRES-UCP1 reporter expression.

### 2.5 UCP1 reporter expression is also found in the ovary and testis.

UCP1 reporter expression was examined in female and male reproductive tissues to assess Cre activity. In the female control mice, the ovary, oviduct and surrounding white adipose tissue did not show UCP1 protein or tdTomato signal (Supplementary Fig. 2, A2-D2). In contrast, the UCP1-IRES-Cre females showed tdTomato labeling within the ovarian tissues (Supplementary Fig. 2, B3), concentrated within the ovarian follicles including cells in the secondary and pre-antral follicles. However, there was no indication of Cre-activity in oocytes, consistent with limited germline recombination (Supplementary Fig. 2E).

In male mice, UCP1 protein was not detected in the testis or caput epididymis in either control or UCP1-IRES-Cre mice (Supplementary Fig. 2, C2-D2). However, UCP1-IRES-Cre males showed robust tdTomato reporter expression throughout the testis and caput epididymis (Sup. Figure 2, D3). Reporter expression in the testis corresponds to cells within the seminiferous tubules and suggested stage-restricted labeling, with no labeling in the basal spermatogonia but strong labeling in elongating spermatids (Supplementary Fig. 2F). Accordingly, use of this model requires careful breeding strategies, and UCP1-IRES-Cre males are not recommended for breeding to avoid unintended germline recombination. Unlike other adipose tissues, the gWAT showed no signs of “pre-beiged” adipocytes and lacked UCP1 protein or tdTomato reporter expression (Supplementary Fig. 2, B2-B3), which aligns with the limited potential for beiging in this adipose tissue depot.

## 3. Discussion

Cre-driver lines generated by BAC transgenesis have been widely used to understand the biological roles of adipose tissue and thermogenic adipocytes. [1, 2]. The utility of these models has been called into question by findings of Cre activity in non-adipose tissues, including the brain and other peripheral tissues [8, 9]. Because hypothalamic circuits may impact thermogenesis and energy balance, any ectopic recombination in the CNS complicates the interpretation of BAT-specific genetic manipulations. These findings underscore the need for improved genetic tools that faithfully reflect endogenous UCP1 expression while minimizing ectopic recombination. In this study, we demonstrate that the UCP1-IRES-Cre knock-in mouse model faithfully labels brown and beige adipocytes, with greatly reduced off-target Cre activity, providing an alternative tool for studying thermogenic adipocyte biology.

We examined the distribution of Ucp1 and Cre activity throughout the body of UCP1-IRES-Cre; tdTomatoAi14 reporter mice. tdTomato reporter distribution and UCP1 protein localization closely corresponded across adipose tissues. Interestingly, tdTomato expression was more pronounced than endogenous UCP1 protein expression in both iBAT transition zones and iWAT. This is consistent with lineage tracing of cells that transiently expressed Ucp1 during development [2]. Numerous lineage-tracing studies have demonstrated that many adipocytes pass through a beige-like state during postnatal development but lose UCP1 expression in adulthood [14, 15]. Postnatal development of iWAT showed beige adipogenesis with strong UCP1-positive staining, consistent with early developmental beiging that is lost in adulthood [16]. Thus, Cre expression throughout the transition zones between core BAT and surrounding WAT and white adipose depots likely reflects “legacy” activation during early development rather than ongoing thermogenic identity. Even so, we note the existence of many “never-beiged” adipocytes that lack a tdTomato signal. Similarly, gWAT exhibited minimal UCP1 protein and tdTomato reporter expression. Previous research supports this finding [17], showing minimal gWAT Ucp1 expression under basal conditions when compared to subcutaneous depots.

Importantly, we observed substantially less ectopic reporter expression in the brain compared to the Tg(UCP1-Cre)Evdr/J line. We confirmed a robust tdTomato signal in the choroid plexus, while only sparse tdTomato labeling was found in scattered neurons of the DMH and PaF, and no UCP1 protein expression was detected in those regions, suggesting potential UCP1 expression during development. This contrasts with Tg(UCP1-Cre)Evdr/J mice, in which strong Cre activity has been observed across multiple brain regions, including the DMH and VMH [9], both key CNS areas involved in regulating energy homeostasis and BAT thermogenesis [20–23]. While Ucp1 mRNA has been detected at low levels in single-cell RNA seq datasets and histological analysis of the choroid plexus [9], the Tg(UCP1-Cre)Evdr/J and Ucp1-CreERT2;NuTRAP strains have demonstrated that Ucp1 promoter activity is not strictly confined to thermogenic adipose tissues [9, 10].

In contrast to the recent characterization of the Tg(UCP1-Cre)Evdr/J line [9], which reported off-target recombination in peripheral tissues including the kidney and adrenal glands, tdTomato reporter expression of our UCP1-IRES-Cre line was largely restricted to adipose tissues. There was sparse reporter expression in the renal medulla and cortex, while other peripheral tissues, including pancreas, liver, heart, and adrenal glands, lacked tdTomato reporter signal. In contrast with Ucp1-CreERT2 mice [10], tdTomato reporter expression was not observed in the iWAT mammary glands UCP1-IRES-Cre mice. The absence of tdTomato reporter expression across metabolically active peripheral tissues supports the conclusion that recombination driven by the UCP1-IRES-Cre line reduces potential confounding effects in future metabolic studies.

Because UCP1-IRES-Cre is a constitutively active Cre line, the tdTomato reporter expression reflects historical Ucp1 promoter activity and cannot be completely distinguished between developmental and active expression, particularly in iWAT and reproductive tissues. Consistent with the recent characterization of Ucp1-iCre mice [11], tdTomato reporter expression was identified in both male and female reproductive tissues. These findings likely reflect low level transient endogenous Ucp1 promoter activity in male germ cells during spermatogenesis, resulting in permanent lineage labeling of elongating spermatids and sperm rather than thermogenic identity [9, 11, 18]. This type of transient activity would result in permanent lineage labeling of elongating spermatids and sperm, not thermogenic activity[17, 19].

This limitation should be considered when designing experiments. However, ectopic recombination is markedly reduced compared to BAC transgenic lines. Off-target recombination in earlier models may have confounded interpretations of BAT-specific genetic manipulations such as food intake, energy expenditure and thermoregulation which are strongly influenced by central neuronal pathways regulating sympathetic communication with adipose tissue [8, 9, 20].

The knock-in design of the UCP1-IRES-Cre places Cre recombinase under the control of the endogenous Ucp1 locus and contributes to the improved tissue specificity observed relative to BAC transgenic models. In conclusion, the UCP1-IRES-Cre line described here supports consistent and specific genetic manipulation of thermogenic adipocytes with minimal confounding effects from ectopic recombination and represents an improved genetic tool for studying thermogenic tissue biology.

## 4. Material and Methods

Mouse generation and husbandry. Mice were bred and housed at Pennington Biomedical Research Center (PBRC) under standard conditions. The UCP1-IRES-Cre mouse line was originally generated in the laboratory of the late Dr. Randall Mynatt at PBRC but has not been previously described in the literature. This mouse line is a non-commercial, investigator-generated mouse line that was originally developed at PBRC. Cryopreserved sperm from the original colony was preserved and later rederived by the PBRC Transgenic Core, after which the line has been maintained through in-house breeding at PBRC and was not obtained from any commercial vendor. No externally owned or client-owned animals were used in this study.

Due to recent findings of widespread ectopic recombination in the hypothalamus of the BAC Tg(UCP1-Cre)Evdr/J model, we recognized the value of this knock-in mouse line for the research community. *Mouse ethics declaration*: All animal experiments were approved by the Institutional Animal Care and Use Committee (IACUC) at Pennington Biomedical Research Center (PBRC). All methods were carried out in accordance with relevant institutional guidelines and regulations for the care and use of laboratory animals. Animals were deeply anesthetized with inhaled isoflurane prior to euthanasia by transcardial perfusion. Animal anesthesia and euthanasia procedures were performed in accordance with American Veterinary Medical Association (AVMA) guidelines. ARRIVE guidelines were followed in all animal experiments.

### Mouse genotyping:

To validate the integrity of the UCP1-IRES-Cre construct, we performed targeted sequencing (Quintara Bioscience, Houston, TX) of the UCP1 locus from a PCR-amplified fragment using the following primers: (FWD: 5’-TGGGCATC TGAAGAGGTAGA-3’; REV: 5’-GCTTGAGGAGAGCCATTTGA-3’). Sequencing results confirmed that the IRES-Cre cassette was inserted immediately downstream of UCP1 exon 6, following the stop codon at the targeted Ucp1 locus (Fig. 1A). Heterozygous UCP1-IRES-Cre (C+; C57BL/6J background) female mice were crossed with homozygous male Rosa-Ai14-tomato^f/f^ (RRID: IMSR_JAX:007914; C57BL/6J background) reporter mice to generate double heterozygous male and female UCP1-reporter (c+) and control (++) littermate mice. Genotyping was performed with PCR on DNA extracted from tail biopsies using the following primers: UCP1–Cre: (Cre FWD: 5’-CGTACTGACGGTGGGAGAAT-3’; Cre REV: 5’-CCC GGCAAAACAGGTAGTTA-3’ U-Cre FWD: 5’-CCTCGGTACACATGCTTTACAT-3’;U-Cre REV:5’-CTGATGGACATGTTCAGGGATC-3’) Ai14 reporter: (Ai14 WT FWD: 5’-AAGGGAGCTGCA GTGGAGTA-3’; Ai14 WT REV: 5’-CCGAAAATCTGTGGGAAGTC-3’; Ai14 Mut FWD: 5’- CTGTTCCTGTAC GGCATGG-3’; Ai14 Mut REV: 5’-GGC ATT AAA GCA GCG TAT CC-3’).

Histology, Immunostaining and Imaging: UCP1-reporter (c+: n = 5; 2 males, 3 females) and control mice (++: n = 5, 3 males, 2 females) were deeply anesthetized with inhaled isoflurane and euthanized by transcardial perfusion with heparinized PBS followed by 4% paraformaldehyde. Peripheral tissues: Bat, iWAT, gonadal WAT (gWAT), heart, liver, pancreas, testes, ovaries and kidney/adrenals) were post fixed, paraffin embedded and sectioned (5 μm). Brains (c+: n = 4; 1 males, 3 females; ++: n = 4, 2 males, 2 females) were cryoprotected in 30% sucrose and sectioned at 30 μm. Immunostaining was performed on Leica Bond RXM automated system (Leica Biosystems) using standard protocols. For primary antibodies we used rabbit anti-RFP antibody (1:300; Rockland, Cat#600-401-379, RRID: AB_2209751) or rabbit anti-UCP1 primary antibody (1:4000 dilution; Abcam Cat# ab209483, RRID: AB_2722676), for detection we used either Alexa Fluor 647 (1:200; Invitrogen, Cat#A32733) or HRP/DAB (ImmPRESS/PACT, Cat#MP-7451, SK-4105, Vector Laboratories), respectively. In peripheral tissues, Hematoxylin Normal (Cat#812, Anatech,Cancer Diagnostics) was used for nuclear stain. In brain, NeuN was stained to visualize anatomical landmarks (mouse anti-NeuN; Millipore Sigma Cat# MAB377; RRID: AB_2298772, 1:1000) and Hoechst (15 ug/ml) was used as a nuclear counterstain in RFP-stained peripheral tissues. Tissues were imaged with the Axioscan 7 slide scanner (ZEISS Research Microscopy Solutions, Carl Zeiss AG, Oberkochen, Germany) and the NanoZoomer Digital Pathology System (Hamamatsu Photonics, Hamamatsu City, Japan). Data were qualitatively assessed for UCP1 and reporter expression, and representative overview and high-magnification images are shown.

## Supplementary Material

This is a list of supplementary files associated with this preprint. Click to download.

- SupplementaryFiguresSR.docx

## Data Availability

Representative data supporting the findings of this study are included within the article and supplementary information files. Additional raw imaging data are available from the corresponding author upon reasonable request.
